# Sjögren’s syndrome and Parkinson’s disease: a bidirectional Mendelian randomization study

**DOI:** 10.3389/fgene.2024.1370245

**Published:** 2024-07-22

**Authors:** Xi Yin, Miao Wang, Fengzhu Li, Zhenfu Wang, Zhongbao Gao

**Affiliations:** Department of Neurology, The Second Medical Center and National Clinical Research Center for Geriatric Disease, Chinese PLA General Hospital, Beijing, China

**Keywords:** Sjögren’s syndrome, Parkinson’s disease, bidirectional Mendelian randomization, causal relationship, genome-wide association studies

## Abstract

**Background:**

Previous epidemiological studies have reported an association between Sjögren’s syndrome (SS) and Parkinson’s disease (PD); however, the causality and direction of this relationship remain unclear. In this study, we aimed to investigate the causal relationship between genetically determined SS and the risk of PD using bidirectional Mendelian randomization (MR).

**Methods:**

Summary statistics for Sjögren’s syndrome used as exposure were obtained from the FinnGen database, comprising 1,290 cases and 213,145 controls. The outcome dataset for PD was derived from the United Kingdom Biobank database, including 6,998 cases and 415,466 controls. Various MR methods, such as inverse variance weighted (IVW), Mendelian randomization Egger regression (MR-Egger), weighted median (WM), simple mode, weighted mode, MR-pleiotropy residual sum and outlier (MR-PRESSO), and robust adjusted profile score (RAPS), were employed to investigate the causal effects of SS on PD. Instrumental variable strength evaluation and sensitivity analyses were conducted to ensure the reliability of the results. In addition, reverse MR analysis was performed to examine the causal effects of PD on SS.

**Results:**

The WM, IVW, RAPS and MR-PRESSO methods demonstrated a significant association between genetically predicted SS and reduced risk of PD (odds ratio OR_WM_ = 0.9988, OR_IVW_ = 0.9987, OR_RAPS_ = 0.9987, OR_MR-PRESSO_ = 0.9987, respectively, *P* < 0.05). None of the MR analyses showed evidence of horizontal pleiotropy (*P* > 0.05) based on the MR-Egger and MR-PRESSO tests, and there was no statistical heterogeneity in the test results of the MR-Egger and IVW methods. The leave-one-out sensitivity analysis confirmed the robustness of the causal relationship between SS and PD. Furthermore, reverse MR analysis did not support any causal effects of PD on SS.

**Conclusion:**

Our MR study supports a potential causal association between SS and a reduced risk of PD. Further extensive clinical investigations and comprehensive fundamental research are warranted to elucidate the underlying mechanisms linking SS and PD.

## Introduction

Parkinson’s disease (PD) is a prevalent and chronic progressive neurodegenerative disorder affecting more than 1% of the population aged >60 years (Simon et al., 2020). PD is characterized by motor symptoms, including resting tremors, bradykinesia, and rigidity, as well as non-motor symptoms, such as cognitive impairment, depression, and sleeping disturbance (Costa et al., 2022). The etiology of PD is complex and cannot be explained by any single cause; instead, PD results from a combination of factors, including mitochondrial dysfunction, disturbance in calcium homeostasis and impairment of protein degradation (Eldeeb et al., 2022). Extensive evidence supports the involvement of inflammation in the onset or progression of PD (Jayaram and Krishnamurthy, 2021). Because of the aforementioned factors, the correlation between PD and autoimmune disorders has emerged as a prominent area of research interest (Freuer and Meisinger, 2022).

Sjögren’s syndrome (SS) is a chronic autoimmune disorder that triggers lymphocytic infiltration and dysfunction of exocrine glands, especially the salivary and lacrimal glands, with symptoms including acute dryness of the eyes, mouth, skin, and mucosa, with accompanying fatigue, arthralgia, neuropathies, and swelling of the salivary glands and lymph nodes (Zhu et al., 2019). Numerous studies have investigated the association between PD and SS(He et al., 2022; Li et al., 2023). Previous research revealed an elevated susceptibility to PD and dementia among individuals with SS compared to the general population (Wang et al., 2022). Additionally, similar results have been reached in other studies that the adjusted hazard ratio (aHR) of developing PD was 1.23 times greater in the SS group than in the non-SS group (Ju et al., 2019; Hsu et al., 2020). However, other studies have produced inconsistent findings indicating that researchers from Sweden and Denmark found no specific association between SS and the risk of PD (Rugbjerg et al., 2009; Li et al., 2012). The potential causal relationship between SS and PD remains a subject of ongoing controversy. Besides, conventional clinical studies might be influenced by confounding factors such as drug intake and sample size. More studies are needed to illuminate the association between SS and PD.

Mendelian randomization (MR) analysis is an emerging epidemiological research methodology that employs genetic instrumental variables (IVs) to evaluate the causality between exposures of interest and outcomes (Burgess and Labrecque, 2018). In MR, IVs are used to investigate the causal association between exposure and outcome by leveraging genetic variants that exhibit strong associations with the exposure and meet specific assumptions (Burgess et al., 2017). Given the random assignment of these variants during conception, the MR approach exhibits reduced susceptibility to confounding factors and potential avoidance of causality bias compared with conventional clinical studies (Sekula et al., 2016; Bell et al., 2021). In the present study, a two-sample bidirectional MR analysis was conducted to investigate the causal effect of SS on PD. Our findings revealed an inverse association between SS and PD, suggesting a potential protective effect of SS against PD progression.

## Materials and methods

### Study design

**FIGURE 1:**
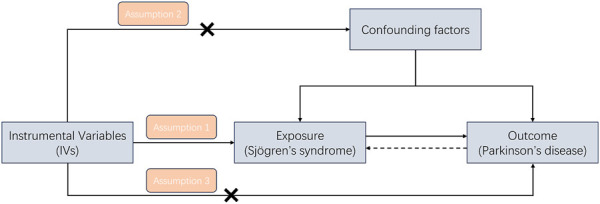
A study design flowchart illustrating the Mendelian randomization (MR) study is presented, with adherence to three essential assumptions required in MR research: (1) the instrumental variables are strongly associated with SS; (2) the instrumental variables are not associated with confounders; and (3) the instrumental variables influence PD only through SS and not through other pathways.

**FIGURE 2:**
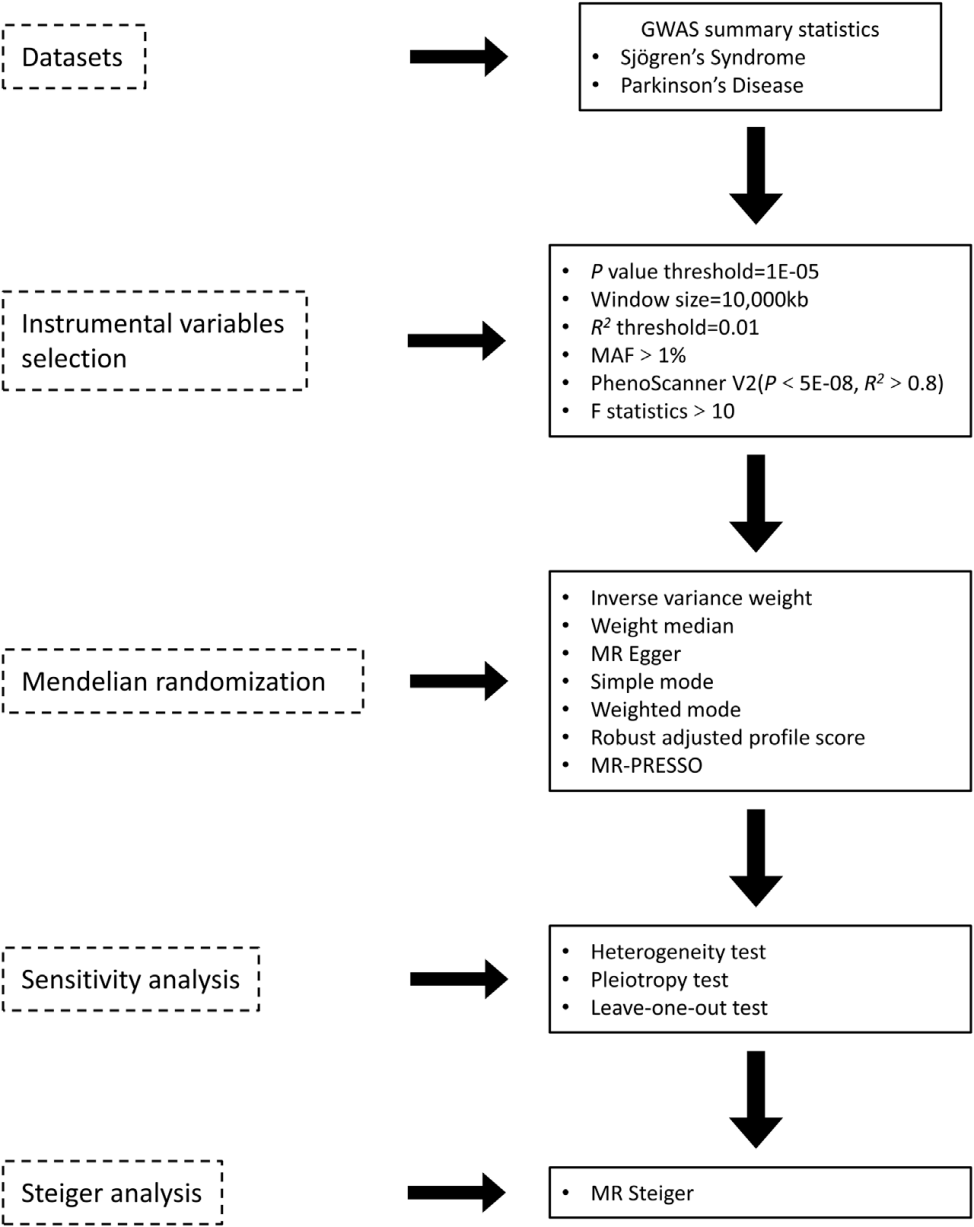
Overview of the two sample MR analyses process in the study. GWAS, genome-wide association study; MAF, minor allele frequency; MR-Egger, Mendelian randomization Egger regression; MR-PRESSO, MR-pleiotropy residual sum and outlier.

An overview of the study design is presented in Figure 1. According to the basic principles of classical MR analysis, three criteria need to be followed in this study: 1) the IVs are strongly associated with SS; 2) the IVs are not associated with confounders; 3) the IVs influence PD only through SS and not through other pathways (namely, horizontal pleiotropy should not be present). The datasets utilized in our study have been made publicly accessible and obtained ethical approval as well as informed consent prior to implementation. This study was conducted on the basis of the latest (STROBE-MR) guidelines (Skrivankova et al., 2021). A flow chart of the study is illustrated in Figure 2.

### Data sources

All data used in this MR study are publicly available. Summary statistics for SS were obtained from the FinnGen database (https://www.finngen.fi/en) (Kurki et al., 2023) and included 1,290 cases and 213,145 controls. For the outcome dataset, the genome-wide association study (GWAS) data for PD were derived from the United Kingdom Biobank database (data set ID: ukb-b-6548). This dataset included 6,998 cases and 415,466 controls of European descent.

### Selection of IVs

To guarantee the validity of the data, a series of quality control techniques were conducted to filter eligible genetic IVs. First, single nucleotide polymorphisms (SNPs) that passed the genome-wide significance threshold (*P* < 1E-08) were chosen as IVs. Because the number of SNPs that met the criteria was too small, we selected IVs with a threshold of *P* < 1E-05 to obtain more comprehensive results. Second, to exclude variants in strong linkage disequilibrium (LD) and ensure the independence of SNPs, the clumping procedure was performed with standard parameters (LD threshold *R* ^2^ < 0.01, window width = 10,000 kb). Third, SNPs with a minor allele frequency (MAF) < 1% were eliminated. Furthermore, the PhenoScanner V2 tool was applied to check for variants associated with other phenotypes (*P* < 5E-08, *R* ^2^ > 0.8) that may affect PD independent of SS (Kamat et al., 2019). For the screened SNPs, the F statistics of SS IVs were calculated to evaluate the strength of the IVs to avoid weak bias (Bowden et al., 2016b). Generally, the calculated result of F statistics >10 indicated a strong correlation between the IVs and exposure, and the MR analysis results avoided weak IV bias (Burgess et al., 2011).

### MR analyses

To evaluate the causal effect of SS on PD, seven MR methods were adopted in our study: the inverse variance weighted (IVW) method (Lawlor et al., 2008; Burgess et al., 2011), Mendelian randomization Egger regression (MR-Egger) method (Bowden et al., 2015; Burgess and Thompson, 2017), weighted median (WM) method (Bowden et al., 2016a; Yin et al., 2022), simple mode method (Walker et al., 2019; Lin et al., 2023), weighted mode method (Hartwig et al., 2017; Li et al., 2024), MR-pleiotropy residual sum and outlier (MR-PRESSO) method (Verbanck et al., 2018; Peng et al., 2022), and robust adjusted profile score (RAPS) method (Zhao et al., 2020; Saunders et al., 2021). Each method makes different assumptions regarding the effectiveness of IVs; therefore, a consistent effect across multiple methods provides the most robust evidence of causal estimates. The IVW method is commonly employed in MR to generate robust causal estimations in the absence of directional pleiotropy. This method computes the weighted average of Wald ratio estimations for each variant, resulting in highly precise estimations when all selected SNPs are valid IVs. The MR-Egger regression method conducts a weighted linear regression making the instrument strength independent of direct effect (InSIDE) presumption that the interconnections between genetic variations and exposure are autonomous of the immediate effects of genetic variations on the result. This method introduces a nuisance parameter to account for potential directional pleiotropy, albeit at the expense of statistical power. Despite its limited statistical capacity, the method offers an estimate that adjusts for multiple effects. The WM approach necessitates that no individual IV accounts for more than 50% of the weight. This approach holds more potency, with a favorable causal impact, especially as the number of unreliable instrumental variables increases. The simple mode method clusters the causal effect estimations for individual SNPs. The causal effect estimate from the largest cluster of SNPs is then adopted as the causal effect estimate. This provides consistent estimates of causal effects under the condition that at least 50% of the IVs are valid. The weighted mode method clusters SNPs into groups based on the similarity of causal effects to estimate the causal effect of the subset with the highest number of SNPs. This method is dependable despite the possibility that most IVs are invalid. The MR-PRESSO method is employed to detect and correct outliers in IVW linear regression. The MR-PRESSO method necessitates that at least 50% of the variants are valid instruments, is balanced in pleiotropy, and depends on the InSIDE condition that instrument exposure and pleiotropic effects are uncorrelated. The RAPS method considers the measurement error in SNP-exposure effects as well as the biases resulting from weak instruments. This method addresses horizontal pleiotropy by relying on robust adjusted contour scores while reducing the deviation caused by horizontal pleiotropy. All of the above methods were applied to comprehensively investigate the causal effects of SS on PD. Moreover, reverse MR analyses were conducted to study the role of PD on the causal effect of SS during bidirectional MR analyses. Finally, the direction of causality for each SNP on exposure and outcome was assessed using MR Steiger, and any SNPs with conflicting outcomes were excluded.

### Sensitivity analyses

In this study, various methods were introduced to conduct sensitivity analyses. MR studies can lead to inaccuracy upon violation of the assumption that IVs are associated only with exposure factors and not confounding variables. To account for this, horizontal pleiotropy was employed to assess the above effect. The intercept term of MR-Egger was used to estimate the possibility of horizontal pleiotropy. In addition, the MR-PRESSO test was conducted to identify and correct potential outlier variants and to evaluate the presence of horizontal pleiotropy. Heterogeneity was assessed by calculating Cochran’s Q value using the MR-Egger and IVW methods. A leave-one-out sensitivity analysis was applied to assess the MR result’s susceptibility to its corresponding IV.

All statistical analyses were performed using the “Two Sample MR” R package, version 0.5.7 (https://mrcieu.github.io/TwoSampleMR/index.html). *p* < 0.05 was considered to indicate a statistically significant result for the MR analyses.

## Results

### Selection of IVs

Twenty-three independent SNPs were identified as instrumental variables of SS following a series of screening procedures. Subsequent analyses were conducted based on the screened SNPs. The F statistics for all IVs exceeded 10, indicating a negligible chance of any weak bias among the independent variables. Detailed information on the IVs is presented in Supplementary Table S1.

### Causal effects of SS on PD

**FIGURE 3:**
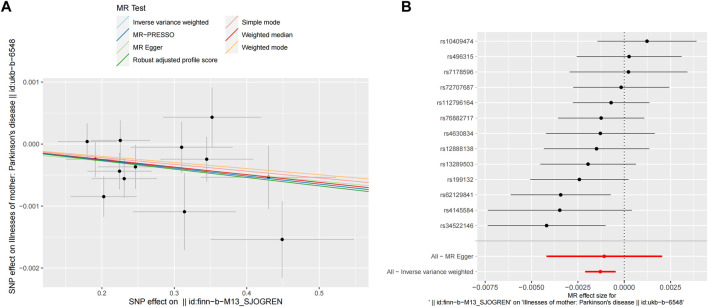
MR analyses result of the causal effect of SS on PD. **(A)** Scatter plot of SNPs potential effects on SS and PD using seven methods. The slope of each line corresponds to the estimated MR effect per method. **(B)** Forest map showing MR analyses results, where each black dot represents a single SNP as IV, shows the logarithm of the OR per SD under the influence of SS; the red dot shows the use of MR-Egger and IVW results of all SNPs; the horizontal line indicates the 95% confidence interval. MR, mendelian randomization; SS, Sjögren’s syndrome; PD, Parkinson’s disease; SNPs, single nucleotide polymorphisms; IV, instrumental variables; OR, odds ratio; SD, standard deviation; MR-Egger, Mendelian randomization Egger regression; IVW, inverse variance weighted.

After removing palindromic SNPs, 13 SNPs were selected to explore the causal effects of genetically predicted SS on PD in the next step analysis. The seven MR methods showed causal effects of genetically predicted SS on PD. Specifically, the WM, IVW, RAPS, and MR-PRESSO methods indicated that genetically predicated SS was significantly associated with decreased risk of PD (OR_WM_ = 0.9988, OR_IVW_ = 0.9987, OR_RAPS_ = 0.9987, OR_MR-PRESSO_ = 0.9987, respectively, all *p* < 0.05). Meanwhile, MR-Egger, simple mode, and weighted mode approaches yielded non-significant results; however, a suggestive inverse association between genetically predicted SS and PD was observed. The scatter plots displayed the single SNP effect and the combined effects of each MR method, and the forest map of IVW supported the inverse relationship between SS and PD onset (Figure 3). Detailed information on the MR analyses of the causal effects of genetically predicated SS on PD was shown in Table 1.

**TABLE 1:** Mendelian randomization estimates for casual effects of SS on PD.

Exposure	Outcome	Method	β	SE	OR	95%CI	*P*-value
Sjögren’s syndrome	Parkinson’s disease	MR Egger	−0.001	0.0016	0.9989	0.9958–1.002	0.511
		Weighted median	−0.001	0.0006	0.9988	0.9977–0.9999	0.026
		Inverse variance weighted	−0.001	0.0004	0.9987	0.9979–0.9995	0.002
		Simple mode	−0.001	0.001	0.9989	0.9969–1.000	0.298
		Weighted mode	−0.001	0.0008	0.9990	0.9974–1.000	0.252
		Robust adjusted profile score	−0.001	0.0004	0.9987	0.9979–0.9995	0.001
		MR-PRESSO	−0.001	0.0004	0.9987	0.9979–0.9995	0.009

### Sensitivity analyses

**FIGURE 4:**
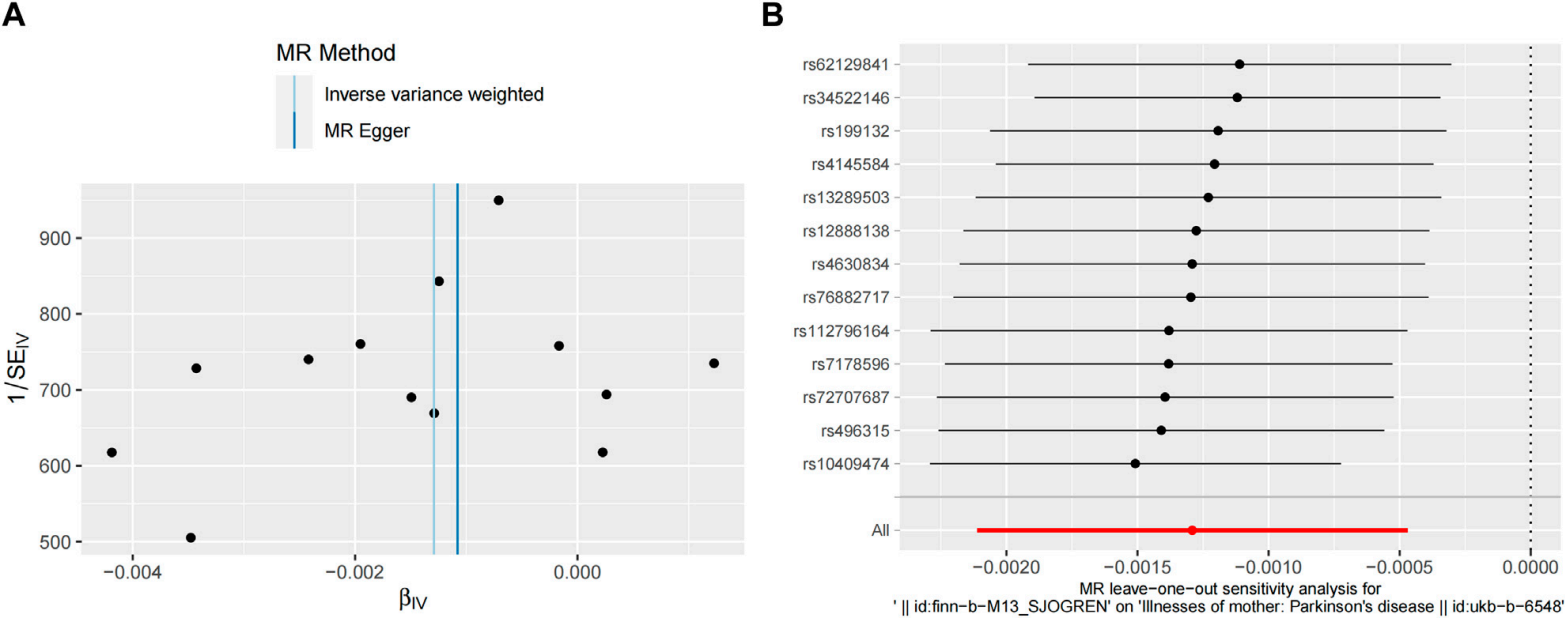
Sensitivity analyses of MR study between SS and PD. **(A)** Funnel plot shows the estimation using the inverse of the standard error of the causal estimate with each individual SNP as a tool. The vertical line represents the estimated causal effect obtained using IVW and MR-Egger methods. **(B)** The leave-one-out method sensitivity analysis. Each black dot represents an IVW method of estimating the causal effect of SS on PD and does not exclude a case where a particular SNP causes a significant change in the overall result. MR, Mendelian randomization; SS, Sjögren’s syndrome; PD, Parkinson’s disease; SNP, single nucleotide polymorphism; IVW, inverse variance weighted; MR-Egger, Mendelian randomization Egger regression.

Pleiotropy, heterogeneity, and sensitivity analyses were performed to enhance the reliability of the results. The MR-Egger and MR-PRESSO global tests showed no horizontal pleiotropy (*p* > 0.05). Furthermore, no statistical heterogeneity was found in the MR-Egger and IVW tests (Table 2). The funnel plots were generally symmetrical, which indicated the reliability of the results. The results of the leave-one-out sensitivity analysis showed that the causal correlation of SS with PD was not driven by a single SNP and that the result was robust (Figure 4).

**TABLE 2:** Pleiotropy and heterogeneity test between SS and PD.

Exposure	Outcome	Pleiotropy test			
		Method	Intercept	SE	*p*
Sjögren’s syndrome	Parkinson’s disease	MR-Egger	−5.78E-05	0.0004	0.892
		MR-PRESSO	NA	NA	0.318
		Heterogeneity test			
		Method	Q	Q_df	Q_*pval*
		MR-Egger	14.291	11	0.217
		IVW	14.316	12	0.281

df, degree of freedom; IVW, inverse variance weighted; Q, heterogeneity statistic Q.

### Reverse MR analyses

**FIGURE 5:**
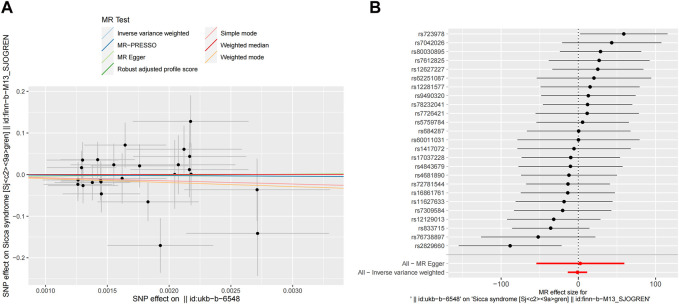
Reverse MR analyses result of the causal effect of PD on SS. **(A)** Scatter plot of SNPs potential effects on PD and SS using seven methods. The slope of each line corresponds to the estimated MR effect per method. **(B)** Forest map showing MR analyses results, where each black dot represents a single SNP as IV, shows the logarithm of the OR per SD under the influence of SS; the red dot shows the use of MR-Egger and IVW results of all SNPs; the horizontal line indicates the 95% confidence interval. MR, mendelian randomization; SS, Sjögren’s syndrome; PD, Parkinson’s disease; SNPs, single nucleotide polymorphisms; IV, instrumental variables; OR, odds ratio; SD, standard deviation; MR-Egger, Mendelian randomization Egger regression; IVW, inverse variance weighted.

To prevent reverse causality from interfering with the above results, a reverse MR study with liability to PD as the exposure and risk of SS as the outcome showed no significant association. The results of all seven MR methods were consistent (Table 3; Figure 5). In addition, no pleiotropy or heterogeneity was found in the reverse MR analyses (Supplementary Table S2). The reliability of the results was demonstrated through funnel plots (Supplementary Figure S1). The directionality of the causal relationship between SS and PD was further confirmed by the MR Steiger test (Supplementary Figure S2).

**TABLE 3:** Reverse Mendelian randomization estimates for casual effects of PD on SS.

Exposure	Outcome	Method	β	SE	OR	95%CI	*p*-val
Parkinson’s disease	Sjögren’s syndrome	MR Egger	2.542	28.992	12.706	2.66E-24–6.06E + 25	0.931
		Weighted median	0.197	8.891	1.2182	3.29E-8–4.51E + 7	0.982
		Inverse variance weighted	−1.247	6.355	0.287	1.12E-6–7.38E + 4	0.844
		Simple mode	−7.542	16.978	0.0005	1.87E-18–1.50E + 11	0.661
		Weighted mode	−9.602	17.454	6.76E-05	9.39E-2–4.87E + 10	0.587
		Robust adjusted profile score	−1.305	6.688	0.271	5.50E-7–1.34E + 5	0.845
		MR-PRESSO	−1.247	6.171	0.287	1.60E-6–5.15E + 4	0.842

## Discussion

In the present study, we employed a comprehensive bidirectional two-sample MR analysis to investigate the causal associations between SS and PD. Our MR findings revealed an inverse relationship between SS and PD, suggesting that individuals with SS have a reduced risk of developing PD.

Our study presented contrasting results to those reported in previous studies. Several researches reported increased odds of PD in patients with autoimmune diseases, especially SS (Wu et al., 2017; Chang et al., 2018; Ju et al., 2019). Patients with SS exhibited a significantly elevated risk of developing PD, as indicated by an adjusted OR of 1.37. In another cohort study, the adjusted hazard ratio (aHR) for PD in individuals with SS was estimated to be 1.23. The evidence of increased risk of PD in individuals with SS has been further substantiated by meta-analyses (Wang et al., 2022; Li et al., 2023). Regarding the underlying mechanisms, it is plausible that both SS and PD share a common autoimmune background. Previous studies have indicated that inflammation plays a pivotal role in the pathogenesis of PD. Inflammatory mediators, such as TNF-α and IL-1β, can induce degeneration of dopaminergic neurons (Tansey et al., 2022). The crucial involvement of proinflammatory cytokines in the pathogenesis of SS is widely acknowledged (Ríos-Ríos et al., 2020). A recent study uncovered the mitochondrial dysfunction in PD and SS patients, revealing the presence of overlapping mitochondria-related genes and mitochondrial DNA damage in these patients (Zong et al., 2024). The aquaporin (AQP) proteins are widely distributed water channels, and the presence of anti-AQP antibodies in SS leads to AQP dysfunction. Dysfunctional AQPs can result in α-synuclein accumulation and activation of astrocytes and microglia, thereby perpetuating inflammation in PD (Sandhya and Danda, 2023). From this perspective, patients with SS may exhibit increased susceptibility to PD, which can be attributed to the potential activation of inflammatory processes within the brain by circulating inflammatory mediators. Nevertheless, prolonged and regular use of immunosuppressive agents in patients with SS has the potential to mitigate neurodegeneration and consequently impede the progression of PD. In line with our findings, patients with rheumatoid arthritis who underwent immunosuppressant therapy have been shown to exhibit a 30% reduction in the risk of developing PD, thereby providing further support for the potential advantageous effects of immunosuppressant therapy in PD development (Rugbjerg et al., 2009). Moreover, in Ju’s research, participants with SS who received non-hydroxychloroquine immunosuppressant therapies showed a lower risk of PD, whereas those using alternative treatments demonstrated an elevated risk (Ju et al., 2019). In addition, laboratory studies have demonstrated that anti-inflammatory drugs effectively diminish the production of neurotoxic molecules and impede the activation of transcription factors governing the expression of genes implicated in immune and inflammatory functions (De Marchi et al., 2023). Collectively, previous findings suggested that the use of medications and other targeted biological agents aimed at mitigating chronic inflammation in patients with SS may contribute, at least partially, to the decreased PD risk observed in our study.

In epidemiological studies, despite investigators employing propensity score matching to address potential confounders such as age, sex, and comorbidities, a consistent presence of bias originating from unknown and unmeasured factors was observed. These deviations have resulted in a reduction in the internal validity of the study. In the present study, one important pitfall was the misdiagnosis of SS and PD. Compared with autoimmune diseases such as rheumatoid arthritis and systemic lupus erythematosus, SS presents with diverse clinical manifestations and lacks well-defined diagnostic criteria, posing challenges with its diagnosis. The diagnosis of PD also poses challenges, with a clinical diagnostic accuracy rate of only 73.8% (Rizzo et al., 2016). The potential misdiagnosis rate may introduce bias into epidemiological studies. MR studies, which evaluate the causal relevance of a risk factor to an outcome, can circumvent the issues of reverse causation and confounding commonly encountered in observational studies (Valdes-Marquez et al., 2019). Seven MR methods were conducted in our study, in which WM, IVW, RAPS, and MR-PRESSO all supported the inverse relationship between SS and PD. In addition, we performed several sensitivity analyses to ensure that all MR assumptions were met to distinguish between a true negative result and the lack of validity of the MR study. Based on the consistent results of MR analyses using different methods, we are confident of the existence of a negative correlation between SS and PD.

Another concern is that the dataset of patients with SS used in our study is of European ancestry. The risk of PD was not specifically associated with SS in a retrospective cohort study conducted in Sweden (Li et al., 2012). Moreover, a case-control study conducted in Denmark revealed no significant correlation between autoimmune rheumatic diseases and PD (Rugbjerg et al., 2009). These conclusions differed from those of studies conducted on the Asian population. The manifestations of autoimmune diseases are significantly influenced by geographical location and ethnic background, encompassing several factors such as genetic predisposition, environmental influences, and interactions between the host and pathogens. Relevantly, previous studies have underscored the existence of geo-ethnic disparities in SS phenotypes (Brito-Zerón et al., 2017; Sandhya and Danda, 2017). The aforementioned epidemiological studies also indicated that the research was restricted to Eastern populations; therefore, the study should be conducted in patients from Western countries (Wu et al., 2017; Chen et al., 2019). The variation observed among different populations reflects the ethnic disparities in disease, which partially accounts for the discrepancies between our research findings and previous studies. Recently, a similar study was conducted, which found no significant causal effect of SS on PD risks based on MR (Wang et al., 2024). Possible explanations for these discrepancies between the two studies include differences in dataset selection and variations in diagnostic rates of SS and PD. Considering these two studies along with previous research, the association between SS and PD remains subject to ongoing discussion. Larger, long-term studies are necessary to further investigate the association between PD and SS as well as elucidate underlying mechanisms.

This study has several limitations that warrant discussion. First, although diverse methods have been employed to investigate diversity, its existence cannot be completely ruled out. Fortunately, consistent results were obtained from multiple analyses, with no evidence of horizontal pleiotropy or heterogeneity detected. Second, our MR study demonstrated a genetically causal relationship between SS and PD; however, the evidence provided by the MR analysis was solely based on genetic factors. Therefore, further clinical studies and experiments are warranted to explore the underlying mechanisms. Third, the participants included in this study were exclusively of European descent, which could induce study population selection bias and pose challenges in elucidating the potential causal association between SS and PD in other populations.

However, our study also has several strengths. First, independent and robust genetic variants were employed as IVs to mitigate the influence of linkage disequilibrium and weak instrument bias. Second, The study had relatively large sample size, allowing us to examine more reliable casual association. Third, seven MR methods were employed to provide robust evidence for the genetic relationship between SS and PD.

## Conclusion

Through MR analyses, our findings provide empirical evidence supporting a potential causal association between SS and a reduced risk of PD. In light of previous studies, the casual association between SS and PD is complicated; therefore, the MR estimates in this study should be interpreted with caution. The underlying mechanism necessitates extensive clinical studies encompassing both Eastern and Western populations, as well as comprehensive fundamental research.

## Data Availability

The original contributions presented in the study are included in the article/Supplementary Material, further inquiries can be directed to the corresponding authors.
