# Supplementary material for: Sjögren’s syndrome and Parkinson’s disease: a bidirectional Mendelian randomization study
Source: Front Genet. 2024 Jul 22;15:1370245. doi: 10.3389/fgene.2024.1370245 (PMC11298492; doi:10.3389/fgene.2024.1370245)
Supplement: Supplementary file 1 [file Table1.docx]

Supplementary table 1. Genetic variants used as instrumental variables.

| SNP | A1 | A2 | EAF | β | SE | p-val | F-statistic |
| --- | --- | --- | --- | --- | --- | --- | --- |
| rs62129841 | T | C | 0.04598 | 0.4489 | 0.0988 | 5.55E-06 | 20.64359459 |
| rs34522146 | A | G | 0.2671 | -0.2025 | 0.0455 | 8.55E-06 | 19.80739041 |
| rs138527475 | G | C | 0.03124 | 0.5556 | 0.1212 | 4.57E-06 | 21.01450838 |
| rs72707687 | G | A | 0.09572 | 0.3101 | 0.07 | 9.44E-06 | 19.6249 |
| rs4145584 | A | T | 0.09126 | 0.314 | 0.0711 | 9.98E-06 | 19.50383861 |
| rs112939612 | C | A | 0.03293 | 0.5455 | 0.1233 | 9.73E-06 | 19.57326864 |
| rs9272305 | G | C | 0.1679 | 0.3364 | 0.0568 | 3.12E-09 | 35.07642333 |
| rs199132 | A | C | 0.285 | 0.2308 | 0.0449 | 2.73E-07 | 26.42280544 |
| rs1131114 | C | T | 0.1823 | 0.3178 | 0.0536 | 3.12E-09 | 35.15427991 |
| rs56025744 | T | C | 0.0588 | 0.3987 | 0.0902 | 9.79E-06 | 19.5379681 |
| rs112796164 | G | T | 0.1107 | 0.3447 | 0.064 | 7.40E-08 | 29.00832275 |
| rs148448202 | G | C | 0.02427 | 0.6301 | 0.139 | 5.80E-06 | 20.54893691 |
| rs13289503 | C | T | 0.7032 | -0.2243 | 0.0441 | 3.67E-07 | 25.86910289 |
| rs76882717 | T | C | 0.05022 | 0.43 | 0.0933 | 4.05E-06 | 21.24093469 |
| rs116898071 | G | A | 0.01057 | 0.9988 | 0.2124 | 2.57E-06 | 22.11301918 |
| rs73212313 | G | A | 0.01944 | 0.6866 | 0.1553 | 9.89E-06 | 19.5463057 |
| rs7178596 | G | A | 0.5623 | -0.1796 | 0.0405 | 9.26E-06 | 19.66539247 |
| rs12888138 | T | C | 0.1892 | -0.2463 | 0.0519 | 2.09E-06 | 22.52133382 |
| rs10409474 | G | C | 0.1044 | 0.3521 | 0.0674 | 1.78E-07 | 27.29054804 |
| rs396568 | A | G | 0.9608 | -0.5257 | 0.1083 | 1.20E-06 | 23.56237376 |
| rs148109864 | T | C | 0.04801 | -0.44 | 0.0967 | 5.33E-06 | 20.70391161 |
| rs4630834 | T | C | 0.4201 | 0.1909 | 0.0408 | 2.97E-06 | 21.89230705 |
| rs496315 | C | T | 0.4992 | 0.2254 | 0.0408 | 3.21E-08 | 30.52020857 |
