# Supplementary figures and images for: Sjögren’s syndrome and Parkinson’s disease: a bidirectional Mendelian randomization study
Source: Front Genet. 2024 Jul 22;15:1370245. doi: 10.3389/fgene.2024.1370245 (PMC11298492; doi:10.3389/fgene.2024.1370245)

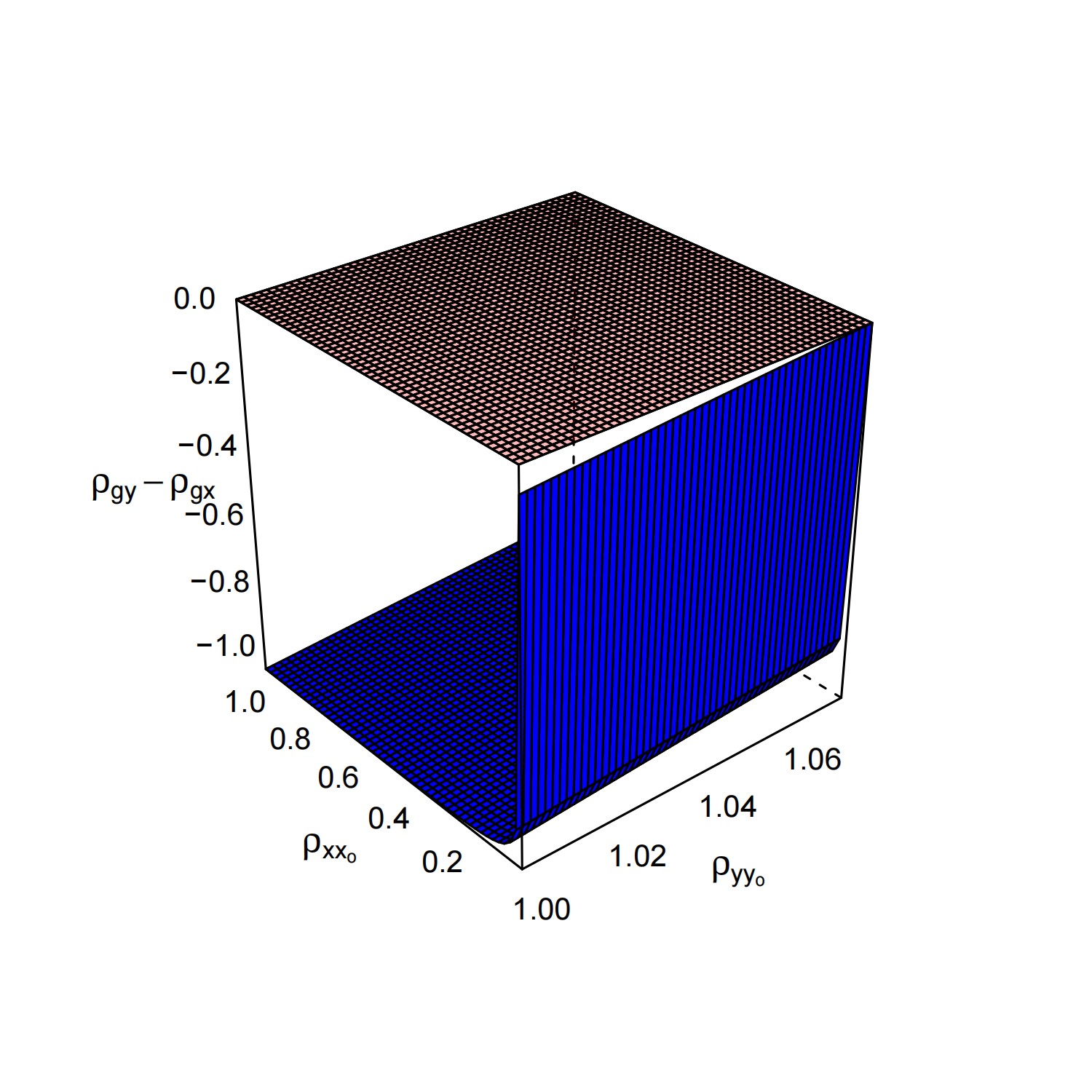

Supplement: Supplementary file 2 [file Image2.TIF]

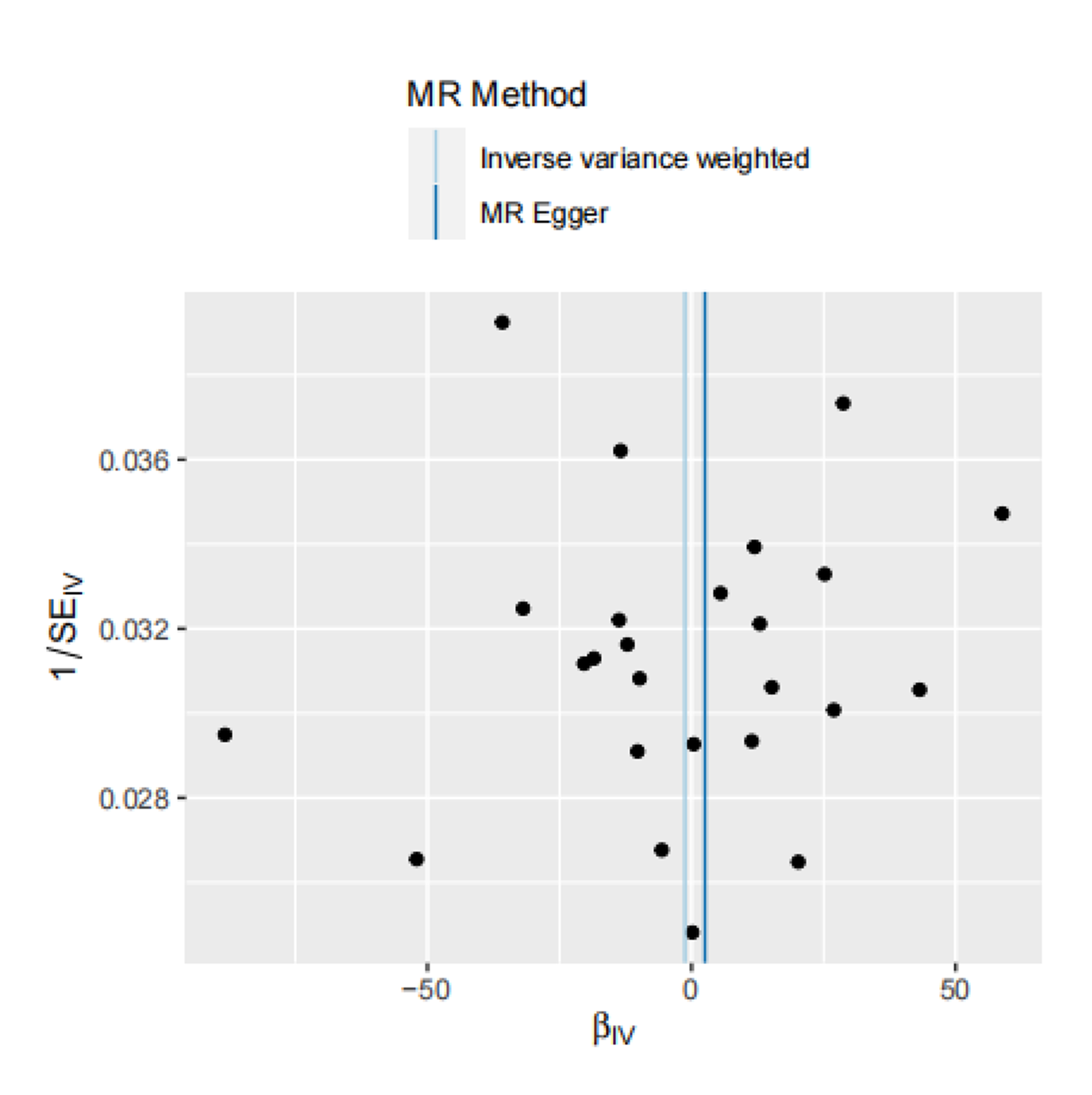

Supplement: Supplementary file 3 [file Image1.TIF]
