# Supplementary material for: Sjögren’s syndrome and Parkinson’s disease: a bidirectional Mendelian randomization study
Source: Front Genet. 2024 Jul 22;15:1370245. doi: 10.3389/fgene.2024.1370245 (PMC11298492; doi:10.3389/fgene.2024.1370245)
Supplement: Supplementary file 4 [file Table2.docx]

| Exposure | Outcome | Pleiotropy test | | | |
| --- | --- | --- | --- | --- | --- |
|  |  | Method | Intercept | SE | *p* |
| Parkinson’s disease | Sjögren’s syndrome | MR-Egger | -0.006 | 0.048 | 0.895 |
|  |  | MR-PRESSO | NA | NA | 0.523 |
|  |  | Heterogeneity test | | | |
|  |  | Method | Q | Q_df | Q_*pval* |
|  |  | MR-Egger | 22.616 | 23 | 0.483 |
|  |  | IVW | 22.634 | 24 | 0.541 |

Supplementary table 2. Pleiotropy and heterogeneity test of reverse MR analysis between PD and

SS.

df，degree of freedom; IVW, inverse variance weighted; Q, heterogeneity statistic Q.
